# Microfluidics-Based Enrichment and Whole-Genome Amplification Enable Strain-Level Resolution for Airway Metagenomics

**DOI:** 10.1128/mSystems.00198-19

**Published:** 2019-05-21

**Authors:** Xing Shi, Changjun Shao, Chunxiong Luo, Yanan Chu, Jian Wang, Qingren Meng, Jun Yu, Zhancheng Gao, Yu Kang

**Affiliations:** a CAS Key Laboratory of Genome Sciences and Information, Beijing Institute of Genomics, Chinese Academy of Sciences, Beijing, People’s Republic of China; b Department of Respiratory and Critical Care Medicine, Peking University People’s Hospital, Beijing, People’s Republic of China; c The State Key Laboratory for Artificial Microstructures and Mesoscopic Physics, School of Physics, Peking University, Beijing, People’s Republic of China; d Center for Quantitative Biology, Academy for Advanced Interdisciplinary Studies, Peking University, Beijing, People’s Republic of China; University of California, Riverside

**Keywords:** emulsion, metagenomics, microfluidic chip, respiratory microbiome

## Abstract

Dysbiosis of airway microbiomes has been found in various respiratory diseases, but its molecular details in terms of taxonomic profile, metabolic characteristics, defensive function, and inhabit adaption are far from clear. Shotgun metagenome sequencing provides detailed information for microbes, whereas its application is rather limited in airways due to host DNA contaminants that overwhelm a minute amount of microbial content. Here, we describe a microfluidics-based enrichment device and an emulsion-based whole-genome amplification procedure (MEEA) for the preparation of DNA from sputa for shotgun sequencing in a metagenomics study. The two protocols coupled in MEEA are first separately assayed with mock samples and are both promising in efficiency and bias. The efficiency and consistency of MEEA are further evaluated in six clinical sputum samples against direct sequencing without enrichment, and MEEA enables 2 to 14 times enrichment for microbial reads, which take 14.68% to 33.52% of total reads. The dominant pathogens detected in MEEA are in excellent agreement with those from clinical etiological tests. Meanwhile, MEEA presents much more microbiome complexity and genome information at a strain level than direct sequencing, exhibiting high sensitivity for identifying prophages and DNA viruses. MEEA provides better microbiome profiling than direct sequencing without a preference for specific microorganisms. The more detailed functional and taxonomic characterization of their species constituents, including both bacterium and virus, facilitates metagenomics studies on the pathogenesis of respiratory microbiomes.

**IMPORTANCE** The airway microbial community, which takes important pathogenic roles for respiratory diseases, is far from clear in terms of taxonomy and gene functions. One of the critical reasons is the heavy contamination of host cell/DNA in airway samples, which hinders the subsequent sequencing of the whole genomic contents of the microbial community—the metagenome. Here, we describe a protocol for airway sample preparation which couples a microbe enrichment microfluidic device and a DNA amplification method performed in numerous droplets. When evaluated with mock and clinical sputum samples, the microfluidics-based enrichment device and emulsion-based whole-genome amplification (MEEA) procedure efficiently removes host cells, amplifies the microbial genome, and shows no obvious bias among microbes. The efficiency of MEEA makes it a promising method in research of respiratory microbial communities and their roles in diseases.

## Abstract (precis)

The airway microbial community, which takes important pathogenic roles for respiratory diseases, is far from clear in terms of taxonomy and gene functions. One of the critical reasons is the heavy contamination of host cell/DNA in airway samples, which hinders the subsequent sequencing of the whole genomic contents of the microbial community—the metagenome. Here, we describe a protocol for airway sample preparation which couples a microbe enrichment microfluidic device and a DNA amplification method performed in numerous droplets. When evaluated with mock and clinical sputum samples, the microfluidics-based enrichment device and emulsion-based whole-genome amplification (MEEA) procedure efficiently removes host cells, amplifies the microbial genome, and shows no obvious bias among microbes. The efficiency of MEEA makes it a promising method in research of respiratory microbial communities and their roles in diseases.

## INTRODUCTION

Accumulating evidence is uncovering the roles of the human microbiome in the pathogeneses of a wide range of diseases, including infectious diseases, tumors, and autoimmune disorders (1–3). Furthermore, the dysbiosis of airway microbiomes has also been found in many respiratory diseases, such as cystic fibrosis, asthma, chronic obstructive pulmonary disease (COPD), and pulmonary fibrosis (4–7). Nevertheless, detailed studies on respiratory microbiomes under pathogenic conditions remain an important pillar of human metagenomics. First, the range of respiratory pathogens is rather broad, including species of pathogenic bacteria, viruses, fungi, and protozoa (8–10). Recent studies have suggested potential effects of bacteriophages on the severity of disease by transferring virulence factor and antibiotic resistance genes (11, 12). A comprehensive study has to challenge the thoroughness and resolution of species detectability in a quantitative way, especially for viruses and bacteria that are commonly codetected in airway samples and occasionally lead to coinfection (13, 14). Second, genetic variations have been found in colonized microbes which generate sister strains coexisting in airway microbiome communities and increase their complexity (15). Distinct lineages or strains of the same species, which carry different functional genes and their variations, may evolve quickly under stresses of the immune system and antibiotics and coinhabit the airway in a mutualistic way (16). Therefore, a successful study of the respiratory metagenome should not only be sufficiently broad to cover species across kingdoms but also sufficiently deep to provide strain-level genomic information for species identification. Among all available techniques, only shotgun metagenome sequencing is able to provide sufficient genome information of microbes and access to such complexity.

Platforms of sequencing technique, both short and long reads, as well as analysis pipelines have flourished in recent years to interrogate pathogenesis mechanisms of the human microbiome (17, 18). As cost keeps dropping, the shotgun sequencing strategy, which provides higher taxonomic resolution and more functional information, has taken the place of 16S rRNA gene amplicon sequencing for studies on the intestinal microbiome (19). However, until now, most studies on airway microbiomes have been based on 16S rRNA gene sequencing. Only a few metagenomic studies have been reported, none of which yielded sufficient raw data to achieve strain-level resolution, and no large-scale clinical studies have been reported. A critical reason for the retardation in the field of respiratory metagenomics is that samples from human airways both are heavily contaminated by host cells/DNA (often up to 96% to 99%) and are in a very limited volume that contain trace amounts of microbial DNA (17, 20–22). Although direct shotgun sequencing of sputum DNA without prior depletion of human cells or DNA is able to provide gross microbiome species profiles and some details for dominant pathogens, such profiling is not sufficiently accurate and only genomic information of the most dominant species is deemed credible due to limited reads of microbes (17, 20–22). Therefore, there is a great need for the development of a methodology that efficiently enriches microbes and amplifies their DNA without bias and allows the identification of species and genes with their original abundances in microbiome samples. Several enrichment methods have been developed, which are categorized into chemical and physical protocols (20, 23–26). The former are largely based on differential lysis between bacterial and host cells when treated with osmotic pressure or lysing agents (25, 26). These kinds of methods are often adequate to remove host cells but are also challenged by biased recovery among bacterial species due to their various sensitivities to the lysing conditions (23, 25). Physical protocols are often based on size selection for microbes, which have much smaller particle sizes (20 nm to 5 μm) than host cells (10 to 40 μm). The large difference between their sizes theoretically ensures more unbiased and efficient selections. However, the efficiency of such methods is often reduced due to severe fluidic structural jam from the large amount of host cells and debris in samples (25). Therefore, a more sophisticated device facilitating high throughput and efficiency is of the essence.

Here, we used a homemade clog-free microfluidic device, which has worked well for enriching pathogen particles from sputum samples (27), to deplete host cell contamination for metagenomics. However, after depletion, the DNA yield of enriched microbes is very low, often in several nanograms that is not sufficient for subsequent library construction and sequencing. To solve this problem, we adopted an emulsion-based whole-genome amplification protocol which performs well in both consistency and accuracy, even for single-cell sequencing, and yields microgram amounts of product from picogram amounts of input (28). The attempt to combine the two procedures, microfluidics-based microbe enrichment and emulsion-based genome amplification (MEEA), has been successful in the preparation of DNA samples for respiratory metagenomics study.

## RESULTS AND DISCUSSION

### Sample preparation and DNA output of MEEA.

**FIG 1:**
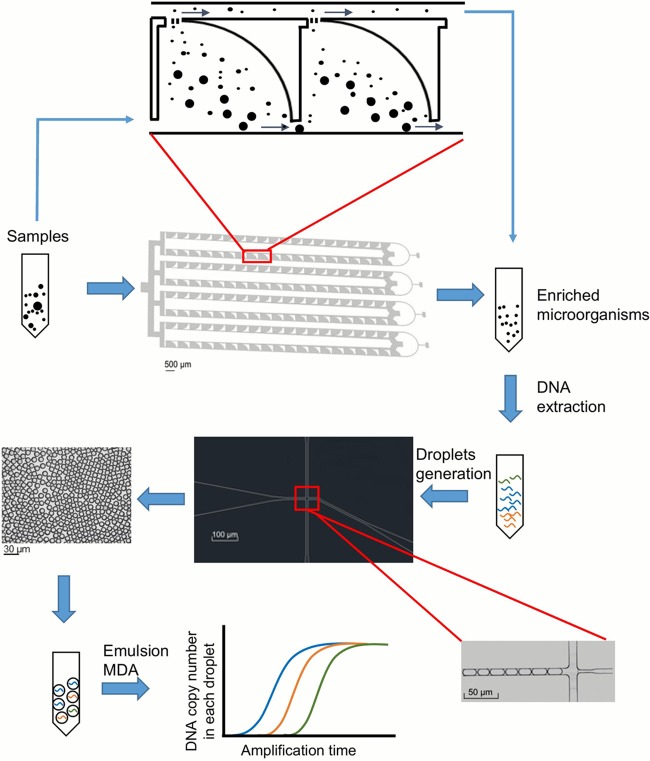
Overview of the MEEA procedure. Preliquefied sputum sample is injected into the inlet of the enrichment microfluidic chip, and enriched microorganisms are collected from the outlets of side channels. Then total DNA is extracted from the solution, added to MDA reaction buffer to a final concentration of 0.5 pg/μl, and distributed in at least 5 × 10^5^ emulsion droplets when going through the microfluidic cruciform. The DNA templates are amplified with MDA in uniformed droplets. After purification, the amplification product is applied to shotgun metagenome sequencing.

The protocol of sputum sample preparation is illustrated in Fig. 1. To obtain at least 500 ng total DNA and more than 10% microbial content, as a rule of thumb for metagenome sequencing, we devised several critical steps to ensure sufficient DNA yields and microbial proportions. The first treatment is fixation before liquefaction. Respiratory samples, such as sputa, are often viscous and require liquefaction to eliminate host cells. A mild fixation with 50% ethanol (see Materials and Methods) protects the integrity of host cells and increases the efficiency of their depletion. The second step is enrichment, where a homemade microfluidic device is used for size selection and is composed of four cascades of 20 repeated selection units, which collects particles less than 5 μm in diameter from a running-through of liquefied sample (27). This design allows high-throughput and clog-free size selection. The injection rate is controlled as fast as 0.5 ml/min, and such a flow rate is applicable for liquefying sputum samples, usually in volumes of 4 to 6 ml; the outputs are often in 1 to 2 ml for each sample. The third step is to achieve consistency in DNA amplification. The enriched sample output, which contains a small number of microbes, usually yields nanograms of DNA after a standard DNA extraction protocol. To obtain sufficient amounts of DNA for sequencing, a multiple displacement amplification (MDA) has been used in single-cell sequencing and often yields micrograms of high-quality DNA (∼20 kb) from picograms of input extracts (29). A previous study has suggested that emulsion-based MDA (eMDA) yields better consistency in DNA amplification, where the length of DNA template in each droplet is optimized to be 20 to 30 kb and 5 × 10^7^ droplets are needed for the preparation of 1 ng DNA (28). We tested three ways to generate droplets for the eMDA: ultrasonic method, interfacial emulsification (30), and cruciform microfluidics (31). Cruciform microfluidics, where droplets are generated when the DNA solution goes across the mineral oil layer at the cruciform, performs the best. The device for this was designed to be scalable and generates droplets of uniform size (10 μm in diameter) with a speed as fast as ∼5 × 10^4^ droplets/min in each of ten parallel channels. The mineral oil was also optimized to be easy for emulsification, to remain stable during amplification, and to not inhibit the activity of polymerase. At the end, the yielded DNA after MEEA from a sputum sample of approximately 1 ml often reaches micrograms in quantity with high quality and an average length of ∼20 kb, and such a preparation is adequate for a shotgun library construction and even for nanopore-based sequencing.

### MEEA efficiently recovers microbes without bias.

**FIG 2:**
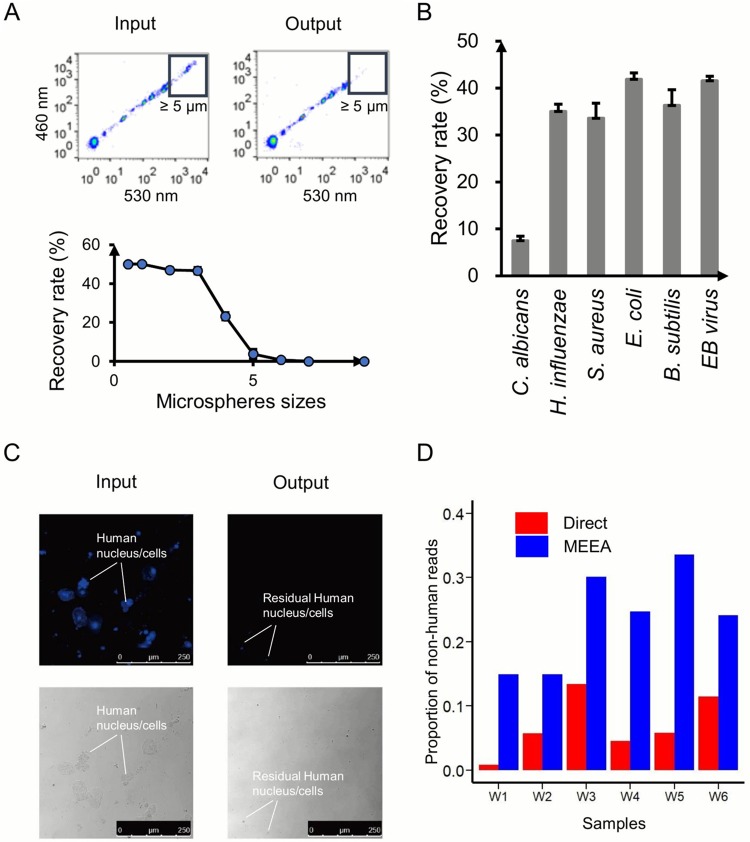
Efficient enrichment of microorganisms without bias. (A) Size selection of microspheres. Thermograms of 0.5- to 9-μm fluorescent microspheres before and after microfluidic chip filter (top) and recovery rates of microspheres of various sizes; error bars indicates means ± standard deviations (SDs) (bottom). (B) The recovery rates of six microorganisms after microfluidic chip filter; error bar indicate means ± SDs. (C) Smears of sputum stained by Hoechst 3342 and viewed under a microscope (×200 magnification) before and after microfluidic chip filter. Blue indicates nuclei and human cells. (D) The proportions of nonhuman DNA in six pairs of sputum samples before and after microfluidic enrichment.

The performance of our enrichment chip was successful in several tests, where both mock and actual sputum samples were used for efficiency and consistency evaluation, especially on size discrimination. First, we mixed fluorescent microspheres with diameters of 0.5, 1, 2, 3, 4, 5, 6, 7, or 9 μm in phosphate-buffered saline (PBS), injected the mixture into the chip, and assayed the input and output with flow cytometry (Fig. 2A). The result shows that microspheres with diameters of >5 μm are rarely collected in the output (Fig. 2A, top), and particles of <3 μm (the size of most bacteria and viruses) have equal recovery rates of ∼50% without bias (Fig. 2A, bottom). Next, we tested the chip with a mock mixture of equal numbers of microbe particles from six microorganisms, including one species of fungus (Candida albicans, 10 to 12 μm), one virus (Epstein-Barr virus, 122 to 180 nm), and four bacteria (Bacillus subtilis, 0.25 to 1 μm by 4 to 10 μm; Escherichia coli, 0.25 to 1 μm by 2.0 μm; Haemophilus influenzae, 0.2 to 0.3 μm by 0.5 to 2.0 μm; and Staphylococcus aureus, 0.5 to 1.0 μm) with various particle sizes. The number of particles was quantified with digital PCR for each species with corresponding primers. As expected, the cells of C. albicans with a diameter of >5 μm did not easily go through the filter, presenting a poor recovery rate of 1.5% to 7.4%, whereas the recovery rates of the four bacteria and Epstein-Barr virus, all with diameters of <3 μm, were roughly the same, ranging from 30.8% to 45.2% (Fig. 2B). Therefore, the present chip is not feasible for most fungi and protozoa, as their cellular sizes are often >3 μm, but is suitable for most bacteria and virus and satisfies the metagenomics studies focusing on them. Finally, the application of MEEA, with subsequent shotgun sequencing, to six clinical sputum samples from patients with acute respiratory infection has given successful results, which are supported by an absence of cells in hematoxylin and eosin (H&E)-stained sample smears by microscopy after the chip filter (Fig. 2C). The proportion of nonhuman reads over all samples was enriched to 14.68% to 33.52% (Fig. 2D), including those with an original proportion of nonhuman DNA as low as 0.99% (patient W1). The higher residual contamination of human DNA is estimated to originate from ruptured host cells, including both mitochondria and nuclei (Fig. 2C). In addition, bacterial cells tend to adhere to mucin fibers, making the efficiency of their separation and enrichment more difficult, and DNase treatment after enrichment shows marginal effects, as the system may be subjected to gross content degradation. A recent study reported that membrane-impermeable DNA intercalators, such as propidium monoazide (PMA), may help chemically deplete the host DNA with high efficiency (25). Although the residual human DNA contamination remains high in our case, we have obtained ∼1-Gb reads of microbes at a common sequencing depth of 4 Gb per sample, which is sufficient for metagenomics analysis of the respiratory microbiome, considering the relatively small biomass. Taking all our results into account, attempts to modulate the present chip design are possible for recruiting more microbes with larger cell sizes, such as fungi and protozoa, but may also increase host contamination; thus, we have decided to keep the original chip dimensions to limit host contamination (27).

### MEEA sequencing provides a path forward for the airway microbiome study.

We next evaluated the efficiency and consistency of emulsion-based metagenome amplification with mock and clinical samples. Our mock DNA was a mixture of extracted DNA from human cells (80%) and bacterial cells (20%; the ratio of E. coli/S. aureus is 100:1). A total of 10 pg of the mixture was amplified with eMDA, followed by pair-end shotgun sequencing (Illumina HiSeq 4000) with a read length of ∼150 bp and a total yield of ∼4 Gb. A comparison of the sequencing result shows that the ratios of the mapped sequencing reads for human/E. coli/S. aureus were 368:125:1 for eMDA and 1,884:114:1 for direct MDA. The latter is severely biased toward host DNA. With eMDA, we have obtained 12 Mb read mapping to S. aureus with a genome coverage of 33.43% and an average depth of 3.26×, implying that genomic information for a single species in 1% relative abundance in microbes (abundance of 0.2% in total DNA sample) can be sufficiently presented.

**FIG 3:**
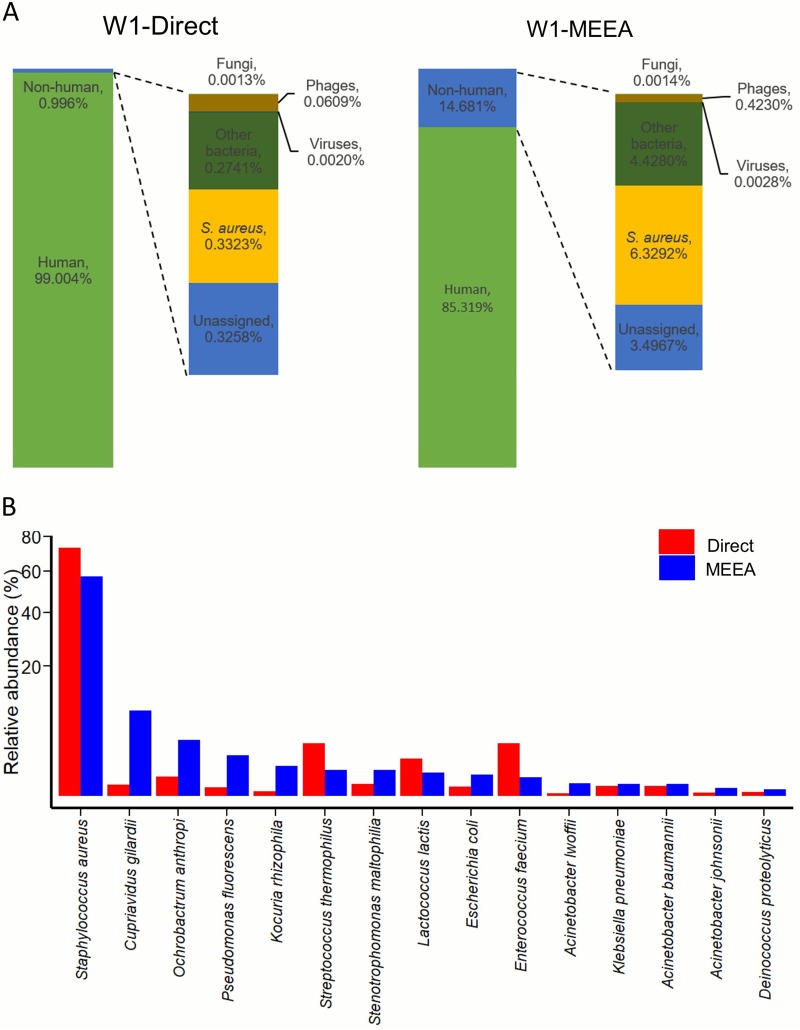
MEEA reveals greater complexity of airway microbiomes in patients with acute infections. (A) Comparison of sequencing results of MEEA and direct sequencing in a typical example of patient W1. (B) The top 15 species with their abundances calibrated as proportions of reads mapped to corresponding reference genome in all nonhuman sequences using bowtie2.

**FIG 4:**
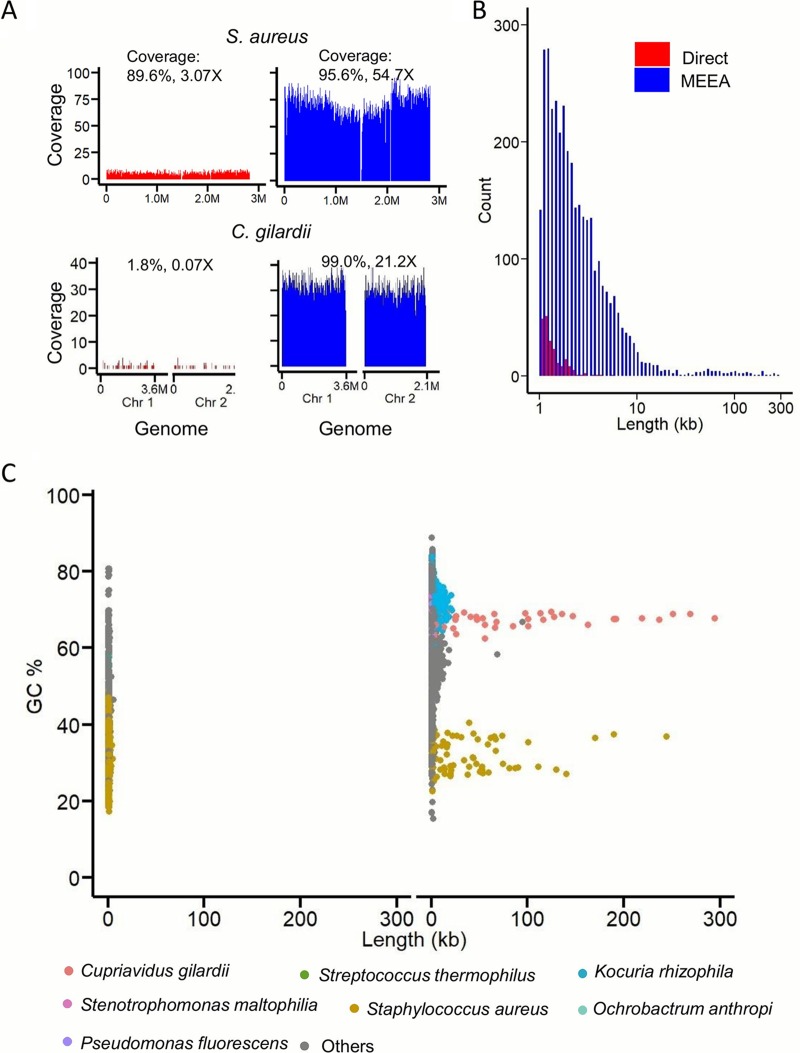
MEEA improves genome assembly. (A) Reads mapped to S. aureus strain V2200 and *C. gilardii* strain CR3. (B) The distribution of lengths of assembled contigs in sample W1; red and blue bars represent direct and MEEA sequencing, respectively. (C) The GC content and length of each contig in direct (left) and MEEA (right) sequencing. Contigs are assigned to major species indicated by colored dots.

We also compared sequencing results of the six clinical sputum samples after the MEEA protocol to those of direct sequencing by using DNA extracted from the original samples. After quality filtration, the clean reads ranged 2.72 to 8.19 Gb in size, with an average of 4.72 Gb, and showed no significant difference between protocols (see Table S1 in the supplemental material). A typical example with an extremely low microbial content is shown and compared between the two protocols in Fig. 3A. The total clean nonhuman reads of the MEEA and direct sequencing protocols were 7.58 million (14.68%) and 0.74 million (0.99%), respectively; most of them were assigned to bacteria. MEEA showed a greater increase in the reads assigned to bacteria and phages than direct sequencing, but did not show preference for fungi (Fig. 3A, Table S1). Both results show the dominance of S. aureus in the original samples, implying that it is the potential causative pathogen. The species profile discovered based on MEEA includes a much broader range of bacterial species (416 species) than the 47 species proposed by direct sequencing (Table S1) when annotated with Kraken. The top 15 species identified with MEEA, which are estimated to take up 96% of bacterial reads assigned by Kraken, were all confirmed by MetaPhlAn, another popular tool for species annotation of metagenomes. However, both Kraken and MetaPhlAn failed to detect all these species in direct sequencing, where Kraken missed six species, including the third-ranked species Ochrobactrum anthropi, and MetaPhlAn missed eleven species, including the second-ranked species Cupriavidus gilardii. We subsequently scrutinized the reads in direct sequencing and found reads mapped to genomes of all the top 15 species detected with MEEA. The low sensitivity of species annotation by the software is mainly due to the small absolute number of reads of these species, in other words, the low sequencing depth obtained with direct sequencing. In addition, the confirmation of major species between the MEEA and direct sequencing data (Fig. 3B) indicates the consistency of MEEA. The increased depth of reads in MEEA brings about a higher genome coverage for most species (Fig. 4A), which greatly promotes not only species annotation but also assembly quality. In MEEA, contig lengths of the assemblies were greatly increased, as well as the number of contigs >500 bp (Fig. 4B). The contig length *N*_50_ values of sample W1 between the direct sequencing and MEEA results were 733 bp and 7,443 bp, respectively. Longer contigs are more informative in gene/genome annotation and interpretation of functional pathways, and there were 478 contigs with a length of >5 kb assembled in the MEEA data but only one in direct sequencing data. The reads mapped to S. aureus are well assembled into a draft of 68 contigs, which is very close to the reference genome of S. aureus strain V2200 with 93.2% genome coverage and 99.57% identity (32). Most of the long contigs were assigned to major species with high abundance, but not always the top one. We also obtained 60 long contigs for the second abundant species *C. gilardii,* covering 99% of its reference genome GCF_001281465.1, which is an opportunistic respiratory pathogen (33) (Fig. 4A and C).

10.1128/mSystems.00198-19.1 TABLE S1 Summary information of reads and species. Download Table S1, XLSX file, 0.01 MB. Copyright © 2019 Shi et al. 2019 Shi et al. This content is distributed under the terms of the Creative Commons Attribution 4.0 International license.

To evaluate the effect of MEEA on functional analysis of metagenomes, we searched antibiotic resistance genes from all the contigs and found 21 in MEEA, which includes all four genes discovered in the direct sequencing data. Among the 21 resistance genes, three nonsynonymous variations in *rpoB*, *parC*, and *murA* genes of S. aureus were identified, which have been reported to confer resistance to rifampin, fluoroquinolones, and fosfomycin, respectively (see Table S2).

10.1128/mSystems.00198-19.2 TABLE S2 Antibiotic resistance genes in sample W1. Download Table S2, XLSX file, 0.01 MB. Copyright © 2019 Shi et al. 2019 Shi et al. This content is distributed under the terms of the Creative Commons Attribution 4.0 International license.

**FIG 5:**
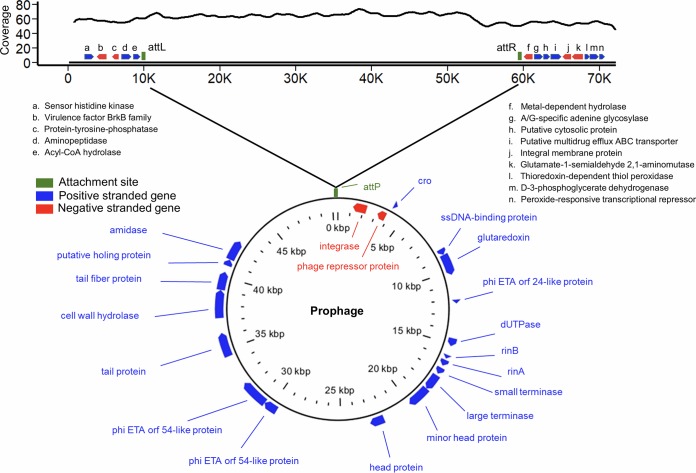
An identified S. aureus prophage. (Top) The contig containing the prophage. Curve above the contig represents the reads coverage, and genes flanking the prophage are indicated with blue (positive-strand gene) and red (negative-strand gene) arrows. (Bottom) A circular genome map of the prophage. Genes in the prophage are labeled above, and green bars represent the attachment site on bacterial genome and prophage.

One of the most prominent advantages of MEEA over the conventional protocol is its increased assembly quality, which allows more efficient prophage discovery. A complete genome of a novel prophage of 50 kb was discovered from sample W1 that was inserted in the genome of S. aureus between an acyl coenzyme A (acyl-CoA) hydrolase gene and a metal-dependent hydrolase gene (Fig. 5). The BLAST result shows that 67.1% of its sequence was aligned to the reference genome of *Staphylococcus* virus phiNM1 (34) with 96.3% identity, and other sequences were mapped to various S. aureus strains and *Staphylococcus* phages. The average read coverage of the prophage was 65.6×, slightly higher than the 58.5× in flanking regions, and such a difference indicates the history of prophage versus phage replications. In addition, two other prophages with partial genomes were also identified on other contigs, and, theoretically, increasing the sequencing depth may further improve the assembly quality and contig length for the exploration of prophages. Prospectively, the emerging nanopore sequencing technology, which is known to provide long sequencing reads (up to ∼800 kb) will certainly promote contiguity in genome assemblies (35) and help to identify large-size mobile elements, such as prophages, genetic islands, and plasmids; the application of our protocols to such efforts is of importance for the study of pathogens and horizontal transfer of resistance and toxic genes.

### Strain-level resolution for dominant species.

As tools for metagenomics with strain-level resolution are emerging rapidly (36–38), the coexistence of distinct strains from the same species in a single sample is usually identified in patients with chronic infections, such as cystic fibrosis and tuberculosis (16, 39). In this work, dominant bacterial pathogens from the six clinical samples were all from a single strain. An exception is patient W2. One of the causative pathogens in this patient was human simplex virus-1 (HSV-1), a eukaryotic virus, occasionally reported as a causative pathogen of pneumonia (40), and we found different strains of HSV-1 coexistent in the patient. It must be mentioned that a large proportion of HSV-1 resides within the host cells and may not pass through the filter in the microfluidic chip, leading to an underestimation of their abundance in the sequencing data. Even though, the reads mapped to HSV-1 cover the complete genome at a depth of 195.63× (compared to the depth of 49.54× in direct sequencing) and help to identify most single nucleotide polymorphisms. The HSV-1 genome sequence from the patient is closest to a published reference strain NC_001806.2. However, 26 high-confidence single-nucleotide variations to this reference, with percentages ranging from 4% to 94%, were found across the 152.2-kb genome (Table 1), indicating a rapid evolution of HSV during the infection. Eleven variants were confirmed by direct sequencing of the original sample, with similar proportions, which means that the sensitivity of detecting variants increases with sequencing depth. These variants occurred in both coding and intergenic regions, and five of them were nonsynonymous coding variations. We noticed that three of the nonsynonymous variants are in RL2, a gene involved in latency (41), and one variation changes the tegument protein that may influence the antigenic property of the virus. Other variations included those in the 5′ untranslated region (UTR) and direct repeat termini. All of them may collectively contribute to the infection or relapse of HSV against the constraint of host immunity and deserve further investigations.

**TABLE 1:** Variant information of HSV-1 in MEEA and direct sequencing from W2

Genomic position	Reference	Variant	Proportion of variant		Annotation	Amino acid change
			MEEA	Direct		
2352	A	G	0.254		ncRNA	
2367	A	G	0.3803	0.381	ncRNA	
8671	T	G	0.188	0.2667	Intergenic	
20592	T	G	0.0945		Intergenic	
24718	C	T	0.9453		Intergenic	
24719	C	T	0.9143		Intergenic	
52423	A	C	0.1		UL26, capsid maturation protease	Synonymous
58440	T	C	0.0845		UL29, single-stranded DNA-binding protein	3′ UTR
76235	G	T	0.2474	0.2045	UL36, large tegument protein	Leu→Leu
79410	T	G	0.6618		UL36, large tegument protein	Synonymous
86871	C	A	0.173	0.1296	UL39, ribonucleotide reductase subunit	Pro→Glu
117703	A	C	0.1308		Intergenic	
118164	A	C	0.0992	0.1875	Intergenic	
122528	T	C	0.0896		RL2, involved in latency	Arg→Gly
124007	T	C	0.4429	0.4167	RL2, involved in latency	Ser→Gly
124022	T	C	0.2188		RL2, involved in latency	Asp→Gly
126820	T	C	0.3667		Intergenic	
134239	T	G	0.4358	0.4444	US2, virion protein	Synonymous
135138	A	G	0.0667		US3, serine/threonine protein kinase	5′ UTR
135144	T	G	0.0809		US3, serine/threonine protein kinase	5′ UTR
138016	C	T	0.8413		Intergenic	
138017	C	T	0.8626	0.8182	Intergenic	
138018	C	T	0.811	0.7273	Intergenic	
139636	T	G	0.0426		Intergenic	
144831	A	T	0.1446	0.375	US11, tegument protein US11	Ser→Thr
144843	G	T	0.4762	0.3333	US11, tegument protein US11	Synonymous

In addition, patient W2, who was attacked by severe community-acquired pneumonia that affected all five lobes, was coinfected by Acinetobacter baumannii and HSV-1 according to the clinical etiological assay. The results are completely consistent with the MEEA sequencing, where A. baumannii was the most dominant species with abundance of 71.3% and HSV-1 with an abundance of 7.2%.

### Conclusions.

The application of our MEEA method leads to gigabases of sequencing reads from airway microbial samples with a limited bias and cost increase. The higher yield and high-quality data ensure more accurate species profiling and gene/genome identification based on better assembly of major species components. Our experience demonstrates that an adequate sequencing depth is crucial for functional analysis, prophage identification, and strain-level resolution, and all these are not easily achievable for the airway microbiome studies at present. Based on size selection, MEEA is able to decontaminate samples of host cells before shotgun sequencing, which makes it applicable to many other complicated host microbiome samples, such as saliva, bronchoalveolar lavage fluid, bronchoalveolar brushings, biopsy samples of intestinal mucosa, and vaginal secretions. The common obstacle of studies with these samples is the trace amount of microbial DNA and severe contamination of host DNA, and MEEA provides a practical method for metagenome sequencing of them, although some adjustments might be needed for each sample type. Furthermore, the MEEA method can be modified for respiratory metatranscriptome sequencing, which will not only include the important missing part of RNA viruses but also validate functional analyses predicted by the metagenome, and paves the way to large-scale shotgun metagenomic studies of respiratory and other complicated microbiomes.

## MATERIALS AND METHODS

### Sample preparation.

A mock sample was prepared comprising six microorganisms: Candida albicans (10 to 12 μm), Bacillus subtilis (0.25 to 1 μm by 4 to 10 μm), Escherichia coli (0.25 to 1 μm by 2.0 μm), Haemophilus influenzae (0.2 to 0.3 μm by 0.5 to 2.0 μm), Staphylococcus aureus (0.5 to 1.0 μm), and Epstein-Barr virus (122 to 180 nm). Equal titers of the six microorganisms in equal volumes were mixed in a suspension. The mock sample for evaluating emulsion MDA was prepared by mixing the genomic DNA of 293T cells (human renal epithelial), Escherichia coli MG1655, and Staphylococcus aureus CH458 at a ratio of 400:100:1. Sputum samples were collected from six patients with acute respiratory infections at Peking University People's Hospital. All study participants provided signed informed consent. The study was approved by the ethics committee at Peking University People's Hospital (number 2016PHB202-01).

### Microfluidic chip preparation.

Two kinds of microfluidic chips were used in this study. One is the enrichment microfluidic chip for which the design is described in our previous study (27), the other is for emulsion according to a previous design (31). The master molds were fabricated using lithography that creates SU8 photoresist (Microchem, Japan) patterns on a silicon wafer. Prepolymer polydimethylsiloxane (PDMS) (Sylgard 184; Dow Corning Toray, Japan) was cast on the silicon mold to a thickness of 5 mm and cured at 70°C for 3 h. Then, the PDMS layer was peeled off and bonded to glass after oxygen plasma treatment and heated overnight at 70°C. Before experiments, holes of inlets and outlets were punched and connected with conduits, and the device was sterilized by UV light and preinfused with sterile PBS.

### Recovery rate testing of microspheres.

The concentrations of green fluorescent polystyrene microspheres (Shanghai Huge Biotechnology Co., Ltd.) of different diameters (0.5, 1, 2, 3, 4, 5, 6, 7, and 9 μm) were adjusted to approximately 1× 10^5^ to 3 × 10^5^ per ml and mixed together. The mixture was injected into the chip, and the output from the side channel and main channel (waste channel) was collected for analysis using a flow cytometer (BD Influx).

### Recovery rate testing of microorganisms.

The mock samples of six microorganisms were filtered through the microfluidic chip. Each of the microorganisms before and after the chip filter was quantified with digital PCR (Bio-Rad QX200); the specific primers and probes of the six microorganisms were synthesized as shown in Table 2.

**TABLE 2:** Primers and probes

Species	Forward primer	Reverse primer	Probe
EB virus^*a*^	CCGGTGTGTTCGTATATGGAG	GGGAGACGACTCAATGGTGTA	TGCCCTTGCTATTCCACAATGTCGTCTT
C. albicans	GATCTCTTGGTTCTCGC	CCCGCCTTACCACTACCG	TCGATGAAGAACGCAGCGAA
B. subtilis	GGAACTGTAACGGCAGCTGATA	CGAACTCGGAAACTCGCATT	TCCTGATCTTCATATCGCGACTCTTGGTG
E. coli	ATCGTGACCACCTTGATT	TACCAGAAGATCGACATC	CATTATGTTTGCCGGTATCCGTTT
S. aureus	AGCATCCTAAAAAAGGTGTAGAGA	CTTCAATTTTMTTTGCATTTTCTACCA	TTTTCGTAAATGCACTTGCTTCAGGACCA
H. influenzae	CCAGCTGCTAAAGTATTAGTAGAAG	TTCACCGTAAGATACTGTGCC	CAGATGCAGTTGAAGGTTATTTAG

a EB, Epstein-Barr.

### Sputum liquefaction.

Sputum samples were liquefied basically as previously described (42). Briefly, sputum was transferred into a sterile centrifuge tube, and for each 1 ml of sputum, 1 ml of 50% ethanol solution, 1 ml of sterilized liquefying solution (6.5 mM dithiothreitol [DTT], 0.15 M sodium chloride, 2.7 mM potassium chloride, 1.4 mM potassium dihydrogen phosphate, 7.8 mM sodium dihydrogen phosphate), and sterilized glass beads of 1 to 5 mm in diameter were added. The tube was vertically rotated at a speed of 20 rpm for 20 min on a rotator. Macroaggregates resistant to liquefaction were further filtered through a 40-μm sterile sieve before the liquefied sputum was applied to the enrichment chip.

### Microfluidic chip enrichment.

Liquefied sputum solution was injected into the microfluidic chip at a rate of 10 ml/h. The output from the side channel was collected and centrifuged at 10,000 rpm for 1 min. The sediment was moved to an Eppendorf tube, and the supernatant was decanted into the Amicon Ultra-4 device (10 kDa; Millipore) and centrifuged at 5,000 rpm for 15 min. The concentrated sample was washed using 2 ml wash buffer three times, and then the concentrate and the above-described sediment were mixed. DNase I (0.05 U/μl; NEB) was added to the resuspended mixture for digestion at 37°C for 30 min and was inactivated at 75°C for 10 min.

### DNA extraction.

DNA from the chip-filtered sample was extracted using a Saliva Genomic DNA kit (Beijing Zoman Biotechnology) according to the manufacturer’s instructions, except that 20 μl S. aureus lysozyme, 40 μl lysate, and 4 μl carrier RNA were first added to the input sample.

### Emulsion MDA.

The emulsion MDA was performed according to the previous study (28). Briefly, the extract DNA was diluted to 0.5 pg/μl in the DNA LoBind tube (Eppendorf) and then added to MDA reaction buffer [50 mM Tris-HCl, 10 mM MgCl_2_, 10 mM (NH_4_)_2_SO_4_, 4 mM DTT, 0.25 μg/μl bovine serum albumin (BSA), 25 μM random primer, 0.5 U/μl phi29 DNA polymerase]. The MDA mixture and FS-D mineral oil (5%; Guangdong Shunde Morsci Biotechnology) were infused into the microfluidic cruciform from separate inlets at an optimized pressure. Emulsion droplets were collected from the output channel and then incubated at 37°C for 20 h for the amplifying reaction. The reaction was inactivated at 65°C for 10 min, and DNA was recovered by adding two volume of chloroform to break the droplets and centrifuged at 13,000 rpm for 10 min to collect the supernatant. Recovered DNA was purified with Agencourt AMPure XP beads (Beckman Coulter) and quantified using a Qubit 2.0 fluorometer (Invitrogen).

### Shotgun sequencing of DNA samples.

The amplified DNA sample was sheared into 300- to 500-bp fragments using an S220 system (Covaris). The PCR-free DNA libraries were constructed with NEXTflex PCR-free DNA sequencing kit (Bioo scientific) according to the manufacturer’s instructions. Shotgun sequencing was performed using HiSeq 4000 (100 bp × 2 for sample W1, 150 bp × 2 for samples W2 to W6) at a depth of 4 Gb per sample at the Beijing Institute of Genomics.

### Data analyses.

**(i) Quality control and decontamination of human sequences.** Adaptor sequences and reads containing ambiguous bases were removed using in-house scripts. Trimmomatic v0.36 (43) was used to trim low-quality reads: 3′ tailing sequences were removed when the average quality over a 4-b sliding window was less than 20, and reads less than 70 bp were discarded. Human reads were filtered by aligning to reference genome GRCh37 using bowtie2 v2.3.4.1 with the option “–very-sensitive-local” (44).

**(ii) Genome assembly and taxonomic annotation.** SPAdes v3.11.0 (45) was used to *de novo* assemble reads to contigs with option “–meta -k 21,33,55,77,” and contigs less than 500 bp were removed. Reads of viruses, phages, and fungi were picked by searching against NCBI nucleotide libraries of viruses and fungi using BLASTN (46) with an E value of <1e−40. Bacterial reads and contigs were assigned to taxa using MetaPhlAn v2.7.6 (47) and Kraken v1.0 (48) simultaneously. A standard bacterial database was used in Kraken, and species abundance was estimated using Bracken (49). The relative abundances of the top 15 species in W1 were calibrated using bowtie2 to align reads to reference genomes of each species.

**(iii) Identification of prophage and antibiotic resistance genes.** Prophages in contigs were identified using PHASTER (50), and the circular genome map of the prophage was generated by Gview (51). Antibiotic resistance genes were identified using RGI in the CARD database with the option “perfect and strict hits only” (52).

**(iv) Genome alignment and SNP calling.** Reads were aligned to reference genome by bowtie2, and single nucleotide polymorphisms (SNPs) were detected using samtools v0.1.19 and bcftools v0.1.19 (53); SNPs with fewer than five reads supported and with a base quality of less than 30 were discarded.

### Ethics approval and consent to participate.

This study was approved by the medical ethics committee of Peking University People's Hospital, Beijing, China (number 2016PHB202-01). Consent was obtained from all patients/guardians.

### Data availability.

The clean sequence data reported in this paper have been deposited in the Genome Sequence Archive in BIG Data Center, Beijing Institute of Genomics (BIG), Chinese Academy of Sciences, under accession number CRA001354.

10.1128/mSystems.00198-19.3 TEXT S1 Script for quality control. Download Text S1, TXT file, 0.04 MB. Copyright © 2019 Shi et al. 2019 Shi et al. This content is distributed under the terms of the Creative Commons Attribution 4.0 International license.
